# Survivin inhibits excessive autophagy in cancer cells but does so independently of its interaction with LC3

**DOI:** 10.1242/bio.037374

**Published:** 2018-10-15

**Authors:** Nicola J. Humphry, Sally P. Wheatley

**Affiliations:** School of Life Sciences, Faculty of Medicine and Health Sciences, Queen's Medical Centre, University of Nottingham, Nottingham NG7 2UH, UK

**Keywords:** Survivin, Birc5, Autophagy, Lysosome, LC3

## Abstract

Survivin expression is pivotal to life and death at the cellular level. For the past decade its pro-survival activity has been attributed to its essential role in cell proliferation and its ability to inhibit apoptosis. However, a growing body of evidence suggests that it may also contribute to cell viability through an as yet undefined role in autophagy. We report that survivin overexpression in osteosarcoma (U2OS) cells is associated with increased LC3-II expression, smaller autophagosomes, enlarged lysosomes and reduced autophagic flux. We also demonstrate that survivin binds LC3 directly through a canonical LC3-interacting region (LIR) in its baculovirus inhibitors of apoptosis protein (IAP) repeat BIR domain, mutation of which inhibits the interaction, but does not abrogate its influence on autophagy. Collectively these data suggest that survivin expression restricts autophagic flux, thereby inhibiting late-stage autophagy and preventing cell death, but does so independently of LC3.

## Abstract (precis)

**Summary:** Survivin binds to LC3 through a canonical LIR, yet this interaction is not required for survivin overexpression to reduce autophagic flux, suggesting that survivin regulates autophagy via an alternative mechanism.

## INTRODUCTION

Implicit in its name survivin is a protein that promotes cell survival. It is overexpressed in cancer (Ambrosini et al., 1997) where its presence correlates with increased resistance to chemotherapy (Paik et al., 2004) and irradiation (Colnaghi et al., 2006), treatments aimed at killing cancer cells. Thus its expression is a biomarker of poor patient prognosis and survivin itself is a promising target for cancer therapy.

Survivin is both essential for mitosis and can suppress cell death (Altieri, 2008; Wheatley and McNeish, 2005). It has a baculovirus inhibitors of apoptosis protein (IAP) repeat (BIR) domain that assigns it membership to the IAP family. Although expression of survivin is cytoprotective (Ambrosini et al., 1997), and its depletion increases apoptosis (Li et al., 1999; Ambrosini et al., 1998), the exact mechanism by which it inhibits cell death remains uncertain. In terms of apoptotic inhibition, several models have been suggested, including inhibition of the mitochondrial apoptosis promoting factor Smac/DIABLO (Pavlyukov et al., 2011; Song et al., 2003); stabilization of X-linked inhibitor of pro-apoptotic protein (XIAP) (Dohi et al., 2004); and induction of the mitochondrial-nuclear translocation of apoptosis inducing factor (Ambrosini et al., 1997).

Recently, several lines of evidence have suggested that survivin may aid the evasion of cell death in ways distinct from apoptosis, one of which is manipulation of autophagy, a catabolic process in which intracellular components are broken down and re-used. Briefly, autophagy targets specific intracellular components for degradation by encasing them in a double-membraned vesicle, called an ‘autophagosome’ that is rich in the human Atg8 homologue, microtubule-associated protein light chain 3 (MAP-LC3 or LC3) bound to phosphatidylethanolamine (PE), LC3-II (Kabeya et al., 2000). Autophagosomes can fuse with endosomes to form amphisomes before fusing either transiently, or completely, with a lysosome to form an autolysosome (Jahreiss et al., 2008). The autophagosome contents are degraded by hydrolytic enzymes that favour an acidic environment before the sub-components are released back into the cytoplasm. Nascent lysosomes can reform from the autolysosome by a process of budding termed ‘autophagic lysosome reformation’ (ALR) (Yu et al., 2010). Basal levels of autophagy are required for cellular homeostasis but, under stress, including radiation treatment, autophagy is induced to promote cell survival. However, intracellular re-cycling cannot continue *ad infinitum* and excessive autophagy ultimately results in cell death (Maiuri et al., 2007).

Emerging data point to several aspects of autophagy that may involve survivin. Firstly, it co-immunoprecipitates with the key autophagic protein, LC3B (Roca et al., 2008); secondly, its expression is upregulated by the autophagic suppressor, mTOR, within the PI3K/Akt pathway (Roca et al., 2008; Zhao et al., 2010); and finally, it interacts with the autophagic regulators Beclin 1 (Niu et al., 2010) and Atg5 (Maskey et al., 2013). Using live fluorescence imaging, immunoblotting and immunoprecipitation assays, here we report that overexpression of survivin increases LC3-II levels, reduces autophagosome size, enlarges lysosomes, and causes an overall reduction in autophagic flux. We also show that expressing a mutant version, survivin^F61AL64A^, that cannot bind to LC3, yields similar outcomes. Thus, in addition to its roles in mitosis and apoptosis, we show that survivin can also act in a pro-survival manner by preventing excessive autophagy, but it does so independently of its interaction with LC3.

## RESULTS AND DISCUSSION

When survivin is overexpressed in cancer it is hugely detrimental to human health: its abundance correlates with tumour resistance to radiation, and this is recapitulated in cell culture (Colnaghi et al., 2006; Connell et al., 2008; Chakravarti et al., 2004). X-irradiation kills cells by inducing DNA damage and apoptosis, but it also promotes autophagy (Jin et al., 2015; Chen et al., 2015). Therefore we hypothesised that survivin may also protect cells against death by limiting excessive autophagy.

### Survivin reduces autophagic flux

To understand how survivin affects autophagy, we first measured the level of LC3-II in U2OS cells overexpressing survivin with and without chloroquine (CQ). CQ is commonly used to assess autophagic flux (Klionsky et al., 2016): it inhibits late stage autophagy causing accumulation of autophagosomes. High LC3-II levels suggest either increased autophagic flux or inhibition of late-stage autophagy. CQ can help discriminate between these two alternatives as it freely diffuses into lysosomes, but gets trapped there where it inhibits degradative enzymes (Solomon and Lee, 2009; Kunze et al., 1982). If flux is increased CQ will further increase LC3-II levels, however, if late-stage autophagy has been inhibited, no difference will be seen (Klionsky et al., 2016). The use of rapamycin (RAP) in combination with CQ allows us to study autophagic flux above basal levels.

**Fig. 1.**
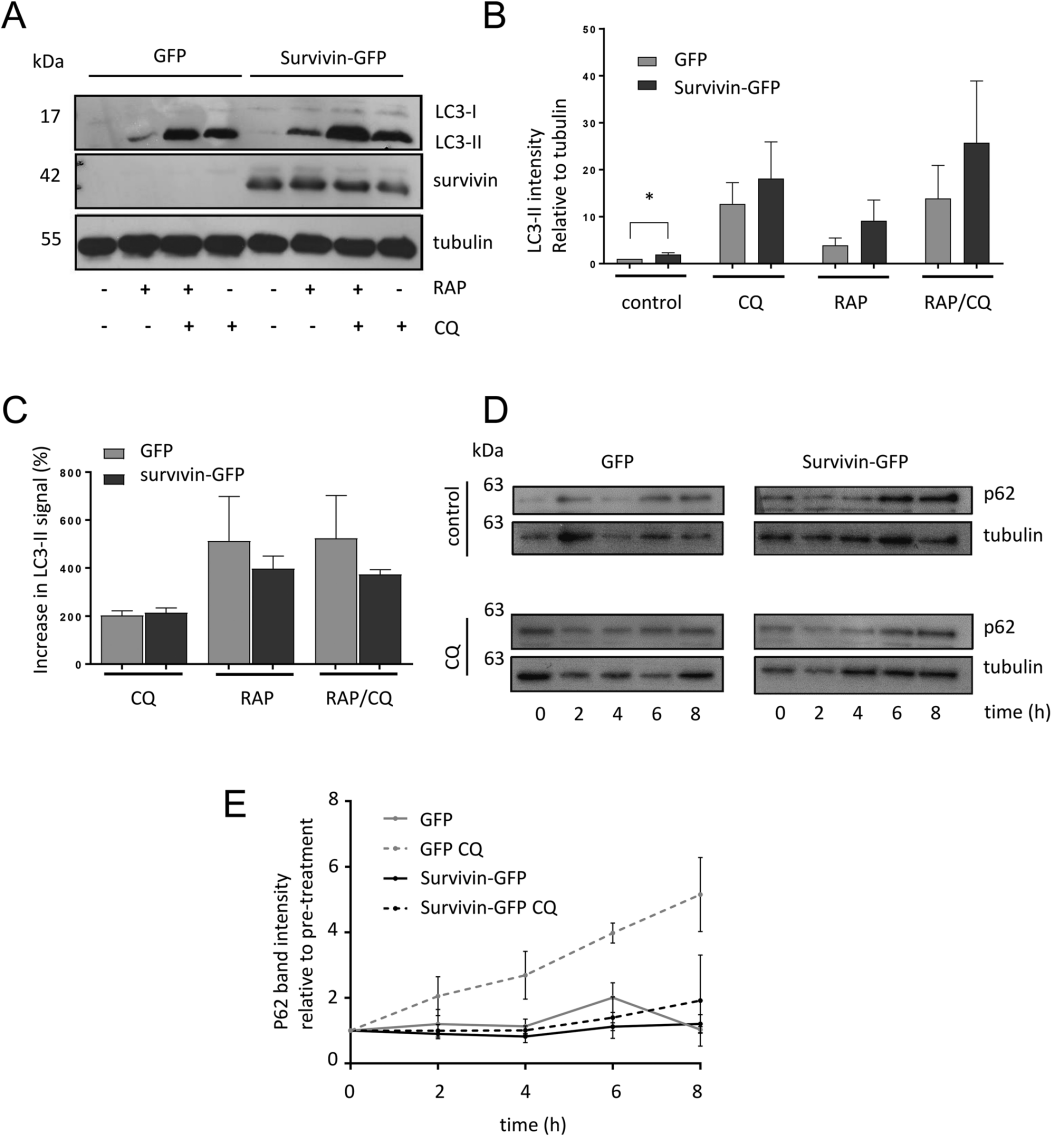
**Survivin regulates autophagic flux.** (A) U2OS cells stably expressing survivin^WT^GFP or GFP alone were treated with RAP (200 nM) and CQ (120 µM) for 2 h, then lysed and immunoblotted with anti-LC3, anti-survivin and anti-tubulin antibodies. Immunoblot shown is representative of four independent experiments. (B) ImageJ quantitation of LC3II signals in (A), normalised against tubulin control and expressed as band intensity relative to untreated GFP cells. (C) Data from (B) expressed as a percentage increase in LC3II between CQ treated and untreated cells to indicate autophagic flux. (D) The above cell lines were treated with CQ (50 µM) for 8 h and p62 levels assessed by immunoblotting at 2 h intervals. Blot shown is representative of three independent experiments. (E) ImageJ quantitation of p62 signals in (D) normalised against tubulin and expressed as band intensity relative to untreated GFP cells. Error bars indicate s.e.m., *N*=3. *P<0.05.

To investigate any potential effects that survivin has on autophagy we chose to use U2OS cells stably expressing survivin^WT^GFP (green fluorescent protein) or GFP (control). These were treated simultaneously with RAP and/or CQ for 2 h, and LC3II abundance quantified by immunoblotting of whole cell lysates (Fig. 1A). Basal levels of LC3-II in survivin^WT^GFP cells were significantly higher than in control cells (Fig. 1B). LC3II levels also appeared higher in survivin^WT^GFP cells after treatment with CQ, RAP or RAP/CQ, however, this did not achieve statistical significance in a Student's *t*-test. To determine whether this was due to induction or inhibition of autophagic flux, we plotted the increase in LC3II signal (Fig. 1C) and found that the increase was similar in both GFP and survivin^WT^GFP cells, thus we concluded that survivin inhibits flux, possibly in the same manner as CQ.

To confirm this finding, we assayed p62 levels in these cells. p62 is an adaptor protein that facilitates autophagic degradation by binding to ubiquitinated targets in the cytosol, and to LC3-II on the autophagosome. As a result, p62 itself is degraded by autophagy and this phenomenon can be used to monitor autophagic flux (Klionsky et al., 2016). U2OS cells stably expressing survivin^WT^GFP or GFP were treated with CQ and p62 expression was assessed at regular intervals from 0-8 h by immunoblotting. In this experiment p62 accumulated significantly more slowly in cells expressing survivin than in control cells, supported by linear regression analysis of treated cell lines (*P*<0.0001; Fig. 1D,E), further substantiating the hypothesis that survivin reduces autophagic flux.

### Survivin decreases the size of LC3-positive puncta

**Fig. 2.**
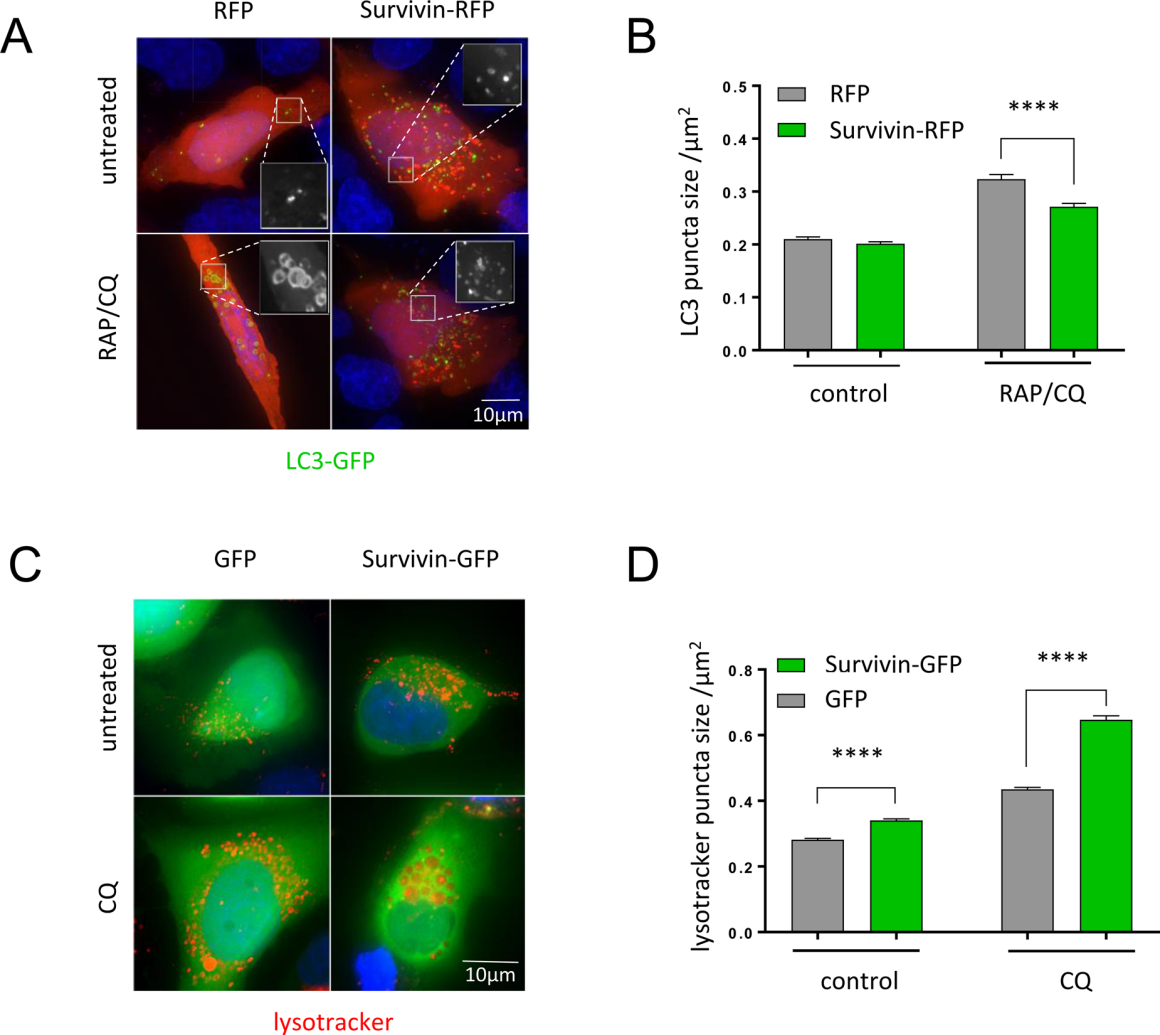
**Survivin regulates autophagosome and lysosome size.** (A) Representative images of U2OS cells transiently expressing survivin-RFP and LC3-GFP treated with RAP (200 nM) and CQ (120 µM) for 2 h then imaged live. (B) Mean GFP-LC3 puncta size was measured using ImageJ, *N*>500 puncta, >35 cells over four independent experiments. (C) Live U2OS cells stably expressing GFP or survivin-GFP were stained with LysoTracker Red to highlight acidic compartments. (D) Mean acidic puncta size was measured using ImageJ, *N*>1500 puncta for>30 cells for each condition in three independent experiments. All error bars indicate s.e.m. ****P<0.0001.

To investigate whether survivin alters the number or size of autophagosomes, we measured these parameters in LC3-positive puncta within the cell before and after RAP/CQ treatment. U2OS cells transiently expressing GFP-LC3 and either red fluorescent protein (RFP) or survivin-RFP were imaged live. RAP/CQ treatment caused an increase in the size of LC3-puncta in both RFP and survivin-RFP expressing cells, but those in cells expressing survivin-RFP were significantly smaller (Fig. 2A,B). The number of autophagosomes was similar in both conditions and increased approximately twofold after RAP/CQ treatment (Fig. S1A). These results suggest that survivin regulates autophagosome maturation, potentially by inhibiting fusion with endosomes (Huotari and Helenius, 2011; Eskelinen, 2005).

### Survivin increases the size of acidic puncta

Fusion of the autophagosome with lysosomes creates an autolysosome with an acidic lumen, allowing lysosomal proteases to digest autophagosomal cell debris. To investigate whether survivin has an impact on lysosomes and autolysosomes, we measured the size and number of acidic puncta within the cell using the cell permeable fluorescent dye, LysoTracker Red, which accumulates in acidic compartments and thus highlights lysosomes and autolysosomes.

To observe the difference in acidic compartments before and after CQ inhibition, U2OS cells stably expressing survivin^WT^GFP or GFP (control) were treated with CQ for 2 h and imaged live using fluorescent microscopy. In both cell sub-lines, CQ treatment increased the average size of acidic puncta, suggesting an accumulation of autolysosomes and a concurrent reduction in nascent lysosomes formation (Fig. 2C,D). Cells expressing survivin^WT^GFP had significantly larger acidic puncta both pre- and post-treatment compared to cells expressing GFP (Fig. 2C,D). No significant difference in the number of acidic puncta between the sub-lines was observed (Fig. S1B), suggesting that like CQ, survivin causes an accumulation of autolysosomes and a concurrent reduction in nascent lysosomes formation. However, since effects were cumulative when survivin overexpression and CQ treatment were combined, this suggests that survivin operates in a different pathway from CQ.

One potential mechanism by which survivin could work is by inhibiting autophagosome-lysosome fusion, as this causes an increase in lysosome (acidic puncta) size (Chen, 2011). Alternatively it may regulate ALR, a process that is triggered by the reactivation of mTOR at the end of the autophagic pathway, which involves the budding of proto-lysosomes from the autolysosome, which then acquire lysosomal hydrolases and mature into lysosomes (Yu et al., 2010). Inhibition of ALR would decrease the number of small, nascent autolysosomes resulting in an increase in mean lysosome size, similar to our observations in survivin-expressing cells.

### Survivin interacts directly with LC3 via a conserved LIR in the BIR domain

Having established that survivin suppresses autophagic flux, restricts the size of LC3 puncta and increases the size of acidic puncta, we next asked whether this inhibition was due to association with LC3, with which it is known to co-immunoprecipitate (Roca et al., 2008).

**Fig. 3.**
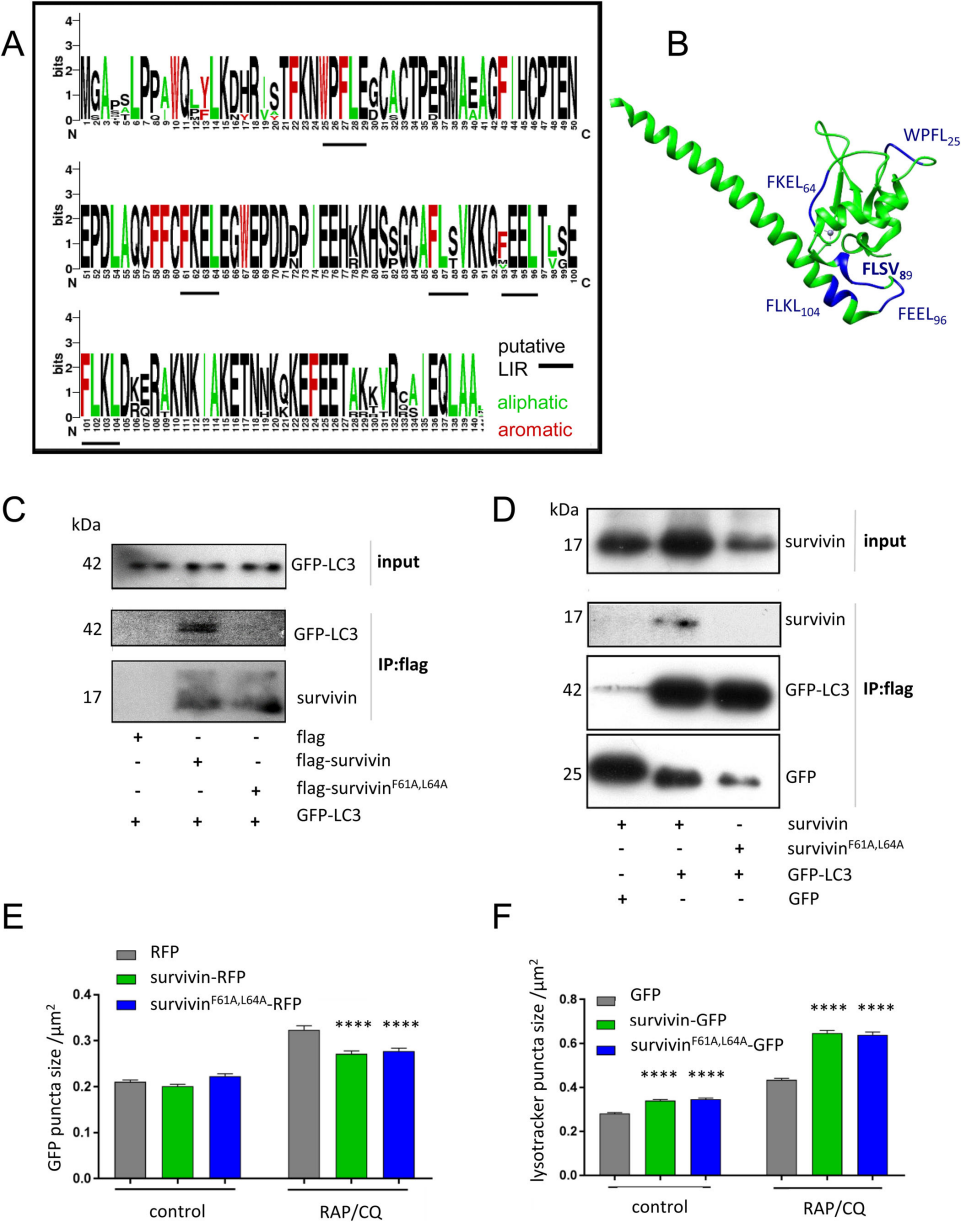
**Survivin F^61^KEL is a canonical LIR but is not required for autophagy inhibition.** (A) The primary sequence of survivin contains five putative LIR motifs, underscored in black within this sequence logo (Schneider and Stephens, 1990) generated from several mammalian homologues of survivin using WebLogo (http://weblogo.berkeley.edu). One stack represents a single position in the sequence, stack height indicates the sequence conservation, and the height of each symbol in the stack indicates the relative frequency of each amino acid at that position. (B) Location of the five putative LIR sites (blue) within the tertiary structure of survivin [PDB 1E31, graphic produced using UCSF Chimera, San Francisco (Pettersen et al., 2004)]. Only F^61^KEL overlaps a beta strand (blue ribbon). (C) HEK293T cells expressing GFP-LC3 were transiently transfected with either Flag, Flag-survivin^WT^ or Flag-survivin^F61A,L64A^. Lysates were immunoprecipitated with anti-Flag antibodies and analysed by immunoblotting for survivin and LC3, *N*=2. (D) HEK293T cells expressing GFP or GFP-LC3 were transiently transfected with survivin^WT^ or survivin^F61A,L64A^. Lysates were immunoprecipitated with anti-GFP antibodies and analysed by immunoblotting for survivin and GFP. (E) U2OS cells transiently expressing RFP, survivin^WT^-RFP or survivin^F61A,L64A^-RFP and LC3-GFP were treated with RAP (200 nM) and CQ (120 µM) for 2 h prior to live cell imaging. Average GFP-LC3 puncta size was measured using ImageJ for each condition *N*>500 puncta over three independent experiments. (F) Living U2OS cells expressing GFP, survivin-GFP or survivin^F61A,L64A^-GFP were stained with LysoTracker Red to highlight acidic compartments and CQ (120 µM) for 2 h, then imaged live. Average size of acidic puncta was measured using ImageJ for each condition, *n*>1400 puncta. All experiments representative of *N*=3. Error bars indicate s.e.m. ****P<0.0001.

The binding of proteins to LC3 usually occurs via canonical LC3 interaction regions (LIR), which are defined as tetrapeptides with an aromatic residue at position one, and a hydrophobic residue at position four: W/F/Y×X L/I/V (where X is any residue) (Noda et al., 2008). Sequence analysis using iLIR (http://repeat.biol.ucy.ac.cy/iLIR/) revealed that survivin has five putative LIR motifs, which are highlighted in the primary sequence and the crystal structure (Fig. 3A,B). The first three of these sequences are located within the BIR domain; the fourth in the central linker between residues 90 and 98; and the fifth at the start of the C-terminal alpha helix. Regression analysis of FASTA alignments (CLUSTAL W2, 10 iterations) of survivin homologues from several mammals (EMBL-EBI: www.ebi.ac.uk), was used to generate a sequence logo using (http://weblogo.berkeley.edu) which showed that all five sites are highly conserved (Fig. 3A). However, since functional LIRs are more frequently associated with exposed beta strands (Noda et al., 2010), we next looked at the position of these five sites in relation to beta strands using UniProt (https://www.uniprot.org/). This stratagem revealed that only F^61^KEL within the BIR domain conforms to this rule, which is highlighted in the blue ribbon (Fig. 3B). Thus we hypothesized that F^61^KEL was a bona fide LIR.

To test this hypothesis, we generated a mutant in which residues at positions one and four of the putative LIR, F^61^KEL, were mutated to an alanine, survivin^F61A,L64A^, tagged with either GFP, RFP or flag, and the immunoprecipitation and fluorescence experiments were repeated. Unlike survivin^WT^, survivin^F61A,L64A^ does not associate with LC3 in either an immunoprecipitation of flag-survivin or an immunoprecipitation of GFP-LC3, clearly demonstrating that this is a functional and unique LIR in survivin (Fig. 3A,B). However, expression of survivin^F61A,L64A^ had the same effect as the wild-type protein on LC3 puncta and acidic puncta size suggesting that interaction with LC3 is not required for these phenomena (Fig. 3E,F; see representative images in Fig. S1-C).

Consistent with this we also noted that there was no significant co-localisation of survivin at LC3-positive puncta suggesting that survivin does not accumulate on the membranes or in the lumen of autophagosomes (data not shown).

Collectively these data demonstrate that survivin inhibits autophagic flux through a mechanism that restricts autophagosome size but it does so independently of its interaction with LC3 and in a manner distinct from CQ. In addition to LC3, survivin interacts with two other proteins involved in autophagosome formation; Beclin 1 (Niu et al., 2010), which is involved in membrane trafficking and localizing autophagic proteins to the phagophore (Kang et al., 2011); and atg5 (Maskey et al., 2013) which extends the phagophore and facilitates LC3 lipidation. Consistent with this, it has been reported that depletion of survivin from erythroblasts causes defects in endosome/lysosomal trafficking, which ultimately manifests as an increase in autophagosomes and can be rescued by expression of vacuolin-1, a protein that promotes endosome-lysosome fusion (Keerthivasan et al., 2012). Survivin has also been shown to bind to HBXIP/LAMTOR5 (Marusawa et al., 2003), a component of the ragulator complex that activates mTOR on the lysosome surface (Bar-Peled et al., 2012), and with clathrin (Keerthivasan et al., 2012) which is included in the membrane of many intracellular vesicles (Royle, 2006) and is also known to regulate ALR (Rong et al., 2012).

We have shown that the inhibitory influence that survivin has on autophagy is independent of its interaction with LC3, which proves that not all LIR motifs and LC3 interactions are autophagy-relevant. So, is there a non-canonical function to the survivin-LC3 interaction? LC3 was initially identified as a protein that interacts with a microtubule-binding protein, and its interaction between a neuronal Ca^2+^-sensor called ‘caldendrin’ appears to be related to microtubule association (Seidenbecher et al., 2004; Mann and Hammarback, 1994). It also binds to FYVE and coiled-coil (CC) domain-containing protein 1 (FYCO1) to mediate microtubule plus-end directed vesicle transport (Pankiv et al., 2010), so one possibility may be that its liaison with LC3 relates to its influence on microtubule dynamics (Rosa et al., 2006). We are currently investigating this possibility.

### Conclusions

In summary, the data herein presented show that survivin can restrict excessive autophagy by reducing autophagic flux and restricting autophagosome maturation. We also identify F_61_KEL as a function LIR in the BIR domain of survivin, and show that its inhibition of excessive autophagy is independent of its interaction with LC3.

## MATERIALS AND METHODS

Unless otherwise stated all reagents were obtained from Sigma-Aldrich.

### Cell culture

Human osteosarcoma cells and human embryonic kidney (HEK 293T) cells were cultured at 37°C, with 5% CO_2_ in Dulbecco's Modified Eagle's Medium (DMEM D6429) supplemented with 10% FCS (Hyclone; PAA Labs). U2OS cells stably expressing GFP or survivin-GFP cells were generated as described in (Jin et al., 2015) and medium supplemented with 50 µg/ml G418 (Geneticin) to maintain expression. Transient transfections were performed using Torpedo transfection reagent (Ibidi) using DNA diluted in PBS as per manufacturer's instructions.

### Plasmid constructs

RFP (pDsRed1), survivin-RFP (pDsRed1, survivin insert), survivinF61AL64A-RFP (pDsRed1, survivinF61AL64A insert), flag (pcDNA3.1, flag insert), flag-survivin (pcDNA3.1, flag-survivin insert), flag-survivinF61AL64A (pcDNA3.1, flag-survivinF61AL64A insert), GFP (pcDNA3.1, GFP insert), survivin-GFP (pcDNA3.1, survivin-GFP insert), survivinF61AL64A-GFP (pcDNA3.1 survivinF61AL64A-GFP), GFP-LC3 (pEGFP-N1, LC3 insert).

### Drug treatments

To induce autophagy cells were treated for 2 h at 37°C with 200 nM RAP (PHZ1235; Thermo Fisher Scientific). Working stocks of 200 µM lyophilized RAP dissolved in DMSO were stored in aliquots at −20°C.

To inhibit autophagic flux, cells were treated with 120 µM CQ (C6628; Sigma-Aldrich) for 2 h at 37°C or 50 µM CQ for >2 h incubation. Working stocks of 100 mM CQ diphosphate salt dissolved in water were stored in aliquots at −20°C.

### Immunoblotting

Standard procedures were used for SDS-PAGE and transfer to PVDF (Amersham Hybond 0.2 µm; GE Healthcare) membrane. Membranes were blocked in 5% (w/v) milk in PBS with 0.1% (w/v) Tween-20. The following primary antibodies were diluted in blocking solution and used to immunoprobe membranes: anti-LC3, which recognises LC3A and LC3B (1:2000; Sigma-Aldrich, L8918), anti-tubulin (1:2000; Sigma-Aldrich, T5168), anti-GFP (1:2000; Sigma-Aldrich, G1546), anti-survivin (1:1000; Novus, NB500-201), anti-histone H3 pT3 (1:2000; 159 Novus, NBP2-61546), anti-p62 (1:1000; Enzo Life Sciences, BML-PW9860). Horse-radish peroxidise-conjugated secondary antibodies (1:2000; Dako) were diluted in either PBS with 0.1% tween (anti-survivin) or blocking medium (all others) and signals detected using enhanced chemiluminescence (Amersham ECL; GE Healthcare) and X-ray film (Amersham Hyperfilm; GE Healthcare). ImageJ (Schneider et al., 2012) was used for quantification.

### Fluorescence imaging

Cells were seeded onto 8-well IbiTreat chambered MicroSlides (Ibidi), incubated overnight, then transfected with relevant DNA constructs, and incubated for 24 h. Medium was replaced with HEPES buffered phenol red free DMEM with 10% FCS supplemented with 200 nM RAP, 120 µm CQ or 0.04 nM LysoTracker Red (L7528; Thermo Fisher Scientific) where applicable. Cells were imaged on an inverted fluorescence microscope (Olympus Delta Vision Elite) using oil immersion objectives: 60× (NA 1.42), or 100× (NA 1.40). Z-stacks were taken at 0.3 µm intervals, deconvolved and z-projected for maximum intensity using SoftWorx software (Applied Precision). Bespoke macros were used with ImageJ (Schneider et al., 2012) software to count and measure puncta.

### Immunoprecipitations

HEK293T cells were used for all co-immunoprecipitation experiments. For immunoprecipitation of survivin, cells were seeded onto a 6-well plate and once approximately 70% confluent, transfected with plasmid constructs for GFP-LC3 and either Flag (control), Flag-Survivin or Flag-survivin^F61A,L64A^. After 24 h, cells were washed with cold PBS and harvested by scraping into ice-cold lysis buffer (50 mM TrisHCl pH 7.5, 150 mM NaCl, 1 mM EDTA, 1% Triton x-100, 1 µg/ml CLAP containing chymostatin, leupeptin, antipain, pepstatin A and 100 µM AEBSF) and lysed on ice (30 min) with gentle pipetting. Lysates were clarified by centrifugation at 17,000 ***g*** to eliminate cell debris and DNA (10 min at 4°C) and the supernatant retained. A sample of the lysate (20 µl) was removed as an input sample and boiled in 20 µl sample buffer (125 mM TrisHCl, pH 6.8, 4% SDS, 20% glycerol, 0.004% bromophenol blue) in a heat block at 90°C for 3 min. To the remainder, Anti-flag M2 Affinity Gel (A2220; Sigma-Aldrich) was added to precipitate flag-tagged proteins as per the recommended protocol.

For immunoprecipitation of LC3, cells were seeded on a six-well plate and grown to 70% confluence, then transfected with plasmid constructs for survivin or survivin^F61AL64A^, and either GFP (control) or GFP-LC3. After 24 h, cells were harvested and re-plated into 10 cm^2^ tissue culture dishes. Once 70% confluent, cells were scraped into 1 ml cold PBS and washed twice by centrifugation at 500× ***g***. Each cell pellet was then resuspended in 200 µl lysis buffer (10 mM Tris/Cl pH 7.5; 150 mM NaCl; 0.5 mM EDTA; 0.5% NP-40, 1 µg/ml CLAP containing chymostatin, leupeptin, antipain and pepstatin A, 100 µM AEBSF) and lysed on ice for 30 min with gentle pipetting*.* DNA and cell debris were pelleted as above and supernatant retained. GFP-Trap-A beads (gta20; ChromoTek) were used to precipitate GFP and GFP-tagged proteins as per the manufacturer's protocol.

### Statistical methods

Data from fluorescence images were analysed using GraphPad Prism where paired immunoblots or unpaired two-tailed *t*-tests were performed to compare the data between conditions, and Welch's correction where sample variance was considered significant by an *f*-test. A *P*<0.05 for the *t*-test or *f*-test was considered significant. Outliers were removed from all fluorescence imaging measurements using the GraphPad ROUT method.

## Supplementary Material

Supplementary information
