# Supplementary material for: Survivin inhibits excessive autophagy in cancer cells but does so independently of its interaction with LC3
Source: Biol Open. 2018 Oct 15;7(10):bio037374. doi: 10.1242/bio.037374 (PMC6215416; doi:10.1242/bio.037374)
Supplement: Supplementary information [file biolopen-7-037374-s1.pdf]

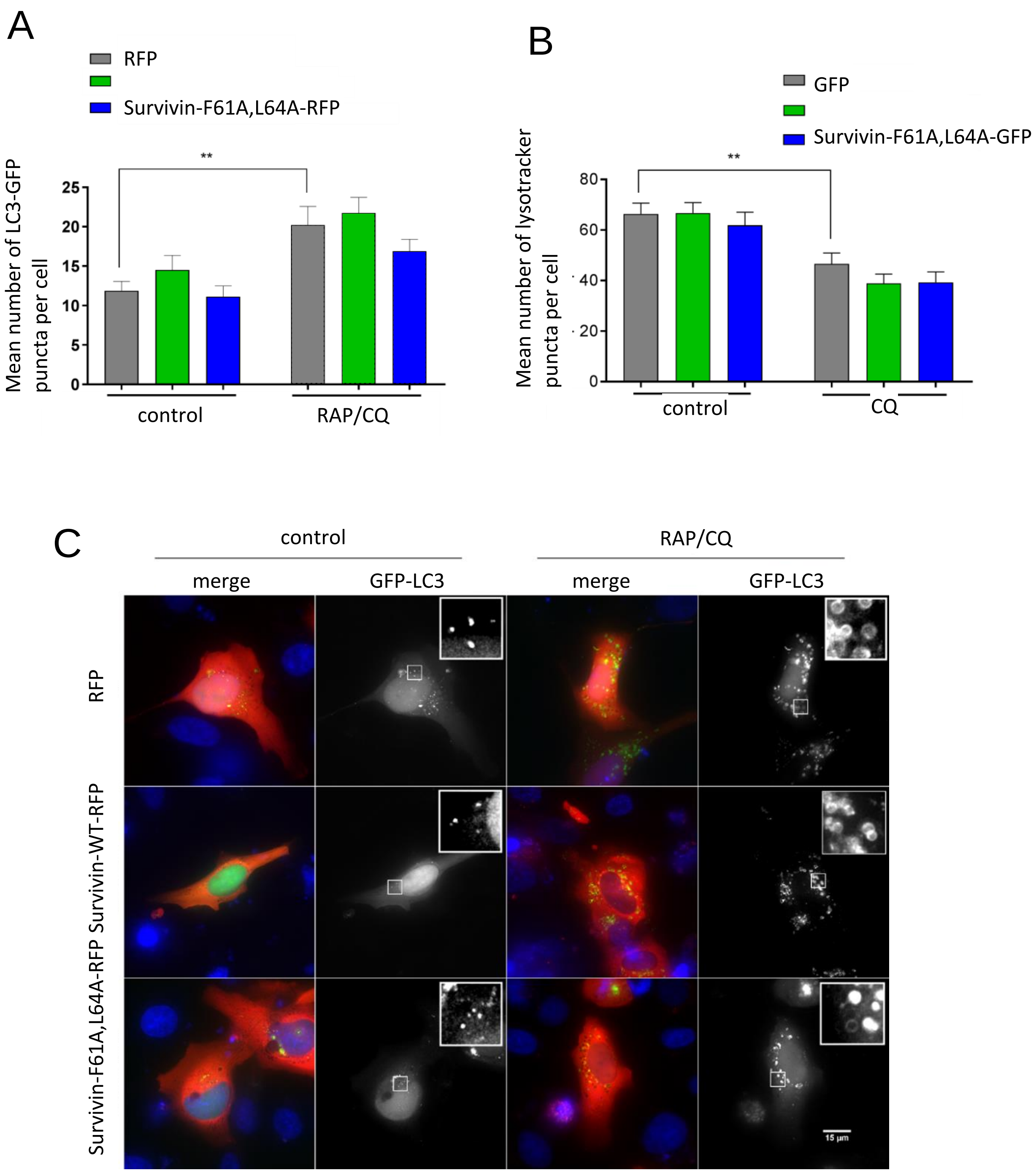

Fig. S1. (A) U2OS cells transiently expressing RFP, survivin-RFP or survivin^{F61A,L64A}-RFP, and LC3-GFP were treated with RAP (200 nM) and CQ (120 µM) for 2h then imaged live. Mean number of GFP-LC3 puncta per cell was assessed per condition, n>35 cells, 3independent experiments. All error bars indicate SEM. **P<0.005, ****P<0.0001. **(B)** Living U2OS cells stably expressing GFP or survivin-GFP were incubated with Lysotracker Red to highlight acidic compartments, and CQ (120uM), for 2h. Mean number of acidic puncta per cell was measured, n>30 cells, 3 independent experiments. Error bars indicate SEM. **P<0.005, ****P<0.0001. **(C)** Representative images of live U2OS cells transiently expressing RFP, survivin-RFP or survivin^{F61A,L64A}-RFP and LC3-GFP treated with RAP (200 nM) and CQ (120 µM) for 2h. n>35 cells, 3 independent experiments. Scale bar 15 µm.
